# From Glomerular Endothelial Remodeling to Kidney Protection: Emerging Molecular Mechanisms and Biomarker Signatures of GLP-1 Receptor Agonists

**DOI:** 10.3390/diagnostics16213645

**Published:** 2026-11-04

**Authors:** Eber Blontrock, Matthijs Oyaert, Risma Ikawaty, Agus Cahyono, Charlotte Delrue, Marijn M. Speeckaert

**Affiliations:** 1 Department of Nephrology, Ghent University Hospital, 9000 Ghent, Belgium; eber.blontrock@ugent.be (E.B.); charlotte.delrue@ugent.be (C.D.); 2 Department of Laboratory Medicine, Ghent University Hospital, 9000 Ghent, Belgium; matthijs.oyaert@uzgent.be; 3 Department of Biomedical Science, Faculty of Medicine, University of Surabaya, Surabaya 60293, Indonesia; risma.ikawaty@staff.ubaya.ac.id (R.I.); agus_jsc@yahoo.co.id (A.C.); 4 Research Foundation-Flanders (FWO), 1000 Brussels, Belgium

**Keywords:** glucagon-like peptide-1 receptor agonists, chronic kidney disease, semaglutide, glomerular endothelium, endothelial remodeling, renal biomarkers, proteomics, kidney fibrosis

## Abstract

Kidney-protective therapies for diabetes mellitus have evolved rapidly in recent years, with glucagon-like peptide-1 receptor agonists (GLP-1RAs) offering renal protection. While some recent clinical trials have shown kidney benefits of these agents that extend well beyond improvements in blood glucose levels and reductions in albuminuria, much is still unknown about the precise molecular mechanisms. In this narrative review, we critically summarize the clinical and mechanistic evidence for GLP-1RA kidney protection, focusing on renal receptor localization, changes in glomerular endothelial cells, inflammatory signaling, mitochondrial dysfunction, and extracellular matrix turnover. We highlight recent results from the FLOW trial, which showed clinically significant kidney benefits with semaglutide, and from the REMODEL trial, which combines renal imaging with single-nucleus RNA sequencing and spatial transcriptomics. Although REMODEL did not demonstrate significant improvements in its coprimary imaging endpoints, exploratory molecular analyses identified treatment-associated changes in glomerular endothelial transcriptional programs and predicted endothelial–immune cell interactions. These findings suggest that microvascular remodeling may contribute to renal protection, although direct glomerular endothelial cell GLP1 receptor expression remains to be confirmed. In experimental systems, the pathways identified also involve control of oxidative stress, inflammasome activation, regulation of mitochondrial function, podocyte health, and fibrosis-related signaling, but their causal role in human kidney disease is yet to be proven. Novel proteomic and extracellular matrix biomarkers, such as tumor necrosis factor receptors, kidney injury molecule-1, and markers of collagen turnover, may help phenotype biological treatment responses beyond classical kidney function and albuminuria measures. Finally, tissue-based molecular phenotyping, combined with circulating and urinary markers, may resolve robust molecular signatures, and separating direct receptor effects from secondary systemic effects will be critical to translating GLP-1RA-mediated kidney benefit into biomarker-based nephrology.

## 1. Introduction

Chronic kidney disease (CKD) is a global health problem with a high burden of cardiovascular comorbidity, early mortality, and a significant socioeconomic burden. Despite improved treatment of CKD, mainly based on renin-angiotensin system (RAS) inhibition, sodium-glucose cotransporter-2 (SGLT2) inhibition, and nonsteroidal mineralocorticoid receptor antagonists, a high residual risk of kidney failure and cardiovascular complications persists, which is due to the presence of many interacting pathological processes involved in the progression of CKD, such as glomerular hypertension, metabolic dysfunction, endothelial damage, mitochondrial malfunction, chronic inflammation, and tissue fibrosis [1,2]. In this new treatment approach, glucagon-like peptide-1 receptor agonists (GLP-1RAs) have become a promising therapeutic class. Originally designed to manage dysglycemia by stimulating glucose-dependent insulin secretion, inhibiting unnecessary glucagon secretion, delaying gastric emptying, and regulating appetite, they have shown meaningful clinical impact on glycemia, body weight, blood pressure, cardiovascular outcomes, and kidney-related endpoints. GLP-1R activation may extend beyond glycemic control and may act directly on pathways that lead to kidney damage. Nevertheless, the extent to which kidney protection is mediated by improvements in systemic metabolic risk factors, as opposed to kidney-specific cellular and molecular mechanisms, remains incompletely understood [3,4,5].

These results were usually observed for secondary or exploratory kidney end-points, often driven by the development of macroalbuminuria rather than true loss of kidney function or kidney failure. The FLOW trial was a breakthrough because it more precisely tested kidney outcomes in 3533 patients with type 2 diabetes and established CKD. Semaglutide reduced the primary composite endpoint of major kidney disease events or cardiovascular death by 24% relative to placebo. In addition, semaglutide reduced the annual rate of eGFR decline, indicating benefit beyond changes in the urine albumin-to-creatinine ratio (UACR) [4]. Nevertheless, including cardiovascular death in the primary composite endpoint and the heterogeneous biological determinants of eGFR decline underscore the need to distinguish clinical efficacy from mechanistic interpretation.

Evidence from populations without diabetes further challenges the traditional view that GLP-1RAs’ kidney benefits are predominantly attributable to improved glycemic control. Although this benefit was partly driven by reductions in incident macroalbuminuria, the findings suggested that GLP-1RA-associated kidney protection may extend to broader cardiometabolic populations [3]. Consistent with this possibility, a randomized trial involving patients with overweight or obesity and non-diabetic CKD demonstrated a substantial reduction in albuminuria following semaglutide treatment [5].

Several potentially additive cellular pathways have been hypothesized to account for the cardiorenoprotective effects of GLP-1RAs, spanning from alterations in renal sodium transporter activity, changes in renal and intrarenal hemodynamic perfusion and glomerular function, via improvement of endothelial function, suppression of pro-inflammatory signals, reduction in oxidative stress, preservation of mitochondrial integrity, and suppression of profibrotic events. Experimental studies suggest roles for nuclear factor kappa-light-chain-enhancer of activated B cells (NF-κB), phosphoinositide 3-kinase/Akt, and adenosine monophosphate-activated protein kinase (AMPK) signaling pathways. These interpretations remain challenging because experimental models of kidney disease differ from human kidney disease, and because cellular localization and functional expression of GLP-1Rs in the human kidney remain unclear. This uncertainty matters when interpreting direct effects on podocytes, tubular epithelial cells, and interstitial cells, because improvements in their biological phenotype may also arise indirectly through vascular, immunological, or systemic metabolic mechanisms [6,7,8].

Another major conceptual step is the growing recognition of the glomerular endothelium as a potential pathway for GLP-1RA-mediated renal protection. Endothelial cell dysfunction may serve as a common node between local inflammatory activation and systemic metabolic dysregulation, both of which can alter glomerular permeability and, in turn, drive tissue remodeling [9]. These findings constitute a biologically plausible link between GLP-1RA treatment and changes in renal microvascular inflammation, without clarifying whether these changes are directly promoted by endothelial GLP-1Rs or whether they are secondary to systemic or intrarenal changes.

These developments also expose a fundamental limitation of conventional approaches to evaluating kidney protection. While albuminuria and eGFR are both clinical tools, neither provides the biological specificity needed to elucidate the cellular pathways underlying treatment response. Combining circulating and urinary biomarkers of endothelial dysfunction, tubular injury, inflammation, and extracellular matrix (ECM) remodeling with transcriptomic, proteomic, and metabolomic analysis may provide a more complete picture of treatment-related kidney protection. Such biomarkers require rigorous validation before they can be interpreted as pathway-specific indicators or predictors of individual therapeutic benefit [10,11,12].

This narrative review critically discusses potential pathogenic pathways involved in GLP-1 RA-associated kidney protection, focusing on kidney receptor pharmacology, glomerular endothelial architectural adaptation, and cross-talk between the microvasculature and tubular cell populations, as well as inflammatory signaling, mitochondrial dysregulation, and fibrotic tissue responses. By using the modulating effects of experimentally induced pathways as a surrogate for direct kidney-specific drug actions, we distinguish clinical effects from observational associations and hypotheses that require causal testing, and show how discoveries of novel pathways and candidate molecular markers can bridge biological mechanisms to kidney endpoints.

## 2. Materials and Methods

The review had three key axes: (i) clinical evidence of GLP-1RAs leading to renal protection; (ii) rigorous knowledge of cellular and molecular events explaining renal protection by GLP-1RAs; (iii) value of advanced molecular phenotyping for new insights. Relevant literature was identified through a targeted, non-systematic approach centered on PubMed/MEDLINE and supplemented by examination of reference lists from relevant publications. The literature considered extended through 10 October 2026. We weighted clinical trials more heavily when assessing treatment benefit. Human physiological responses, receptor localization, expression profile assessment of molecules within tissues, and experimental perturbation studies were used to infer a role of the receptor in question, keeping in mind the importance of apparent systemic and/or intrarenal compensations. Explaining changes in transcript levels, molecular expression, metabolite abundance, or computationally derived signaling networks alone was insufficient to establish activation and causation. Candidate biomarkers were assessed based on the specified context of use (prognostic, predictive, pharmacodynamic), and their potential clinical relevance was judged by their association with routine markers and endpoints. We prioritized albuminuria, estimated glomerular filtration rate (eGFR), sustained decline in kidney function, and kidney failure. Emphasis was placed on analytical reproducibility, biological interpretability, independent validation, and additional clinical benefit. For predictive biomarkers, we distinguished evidence of differential treatment effects by baseline biomarker status from associations with disease progression or treatment-associated biomarker changes. Where mechanistic mediation was proposed, temporal relationships, potential confounding, and the need for formal causal inference or experimental validation were considered.

Where results were inconsistent between studies, comparability was considered, and inconsistencies assessed given study design, population, duration of treatment, formulation of GLP1-RA used, definitions of outcomes, and weaknesses in method. The strength of evidence for clinical outcomes was weighted towards RCTs with sufficient power, and for mechanistic outcomes was weighted towards studies that employed methods validated for definitive receptor localization, such as immunostaining, or experimental approaches that validated receptor-specificity, like receptor-specific knock-down or activation. Incongruities were explained within the context of known clinical results versus emerging mechanistic data that require further validation. The limitations of emerging molecular technologies were also examined, including cell-type representation and sampling variability in kidney tissue studies; differences between transcript abundance and protein activity; computational inference of cell-to-cell communication; and the effects of dietary intake, body composition, renal clearance, and sample processing on circulating or urinary biomarker concentrations. Particular attention was given to the challenges of integrating molecular information across biological compartments and interpreting treatment-associated changes in the absence of direct functional measurements.

## 3. Clinical Evidence for Kidney Protection: What Do the Trials Actually Demonstrate?

While reductions in albuminuria were once considered the main sign of GLP-1RA-related kidney protection, newer evidence indicates that certain agents can also slow sustained loss of kidney function and reduce clinically relevant kidney events. Differences in trial populations, background treatments, kidney endpoint definitions, and statistical power make direct comparisons between studies difficult and limit how specifically their findings can be interpreted [4,13,14].

The earliest signals of kidney benefit came from cardiovascular outcome trials that showed reduced renal outcomes in people with type 2 diabetes and increased cardiovascular risk. In the LEADER trial, liraglutide reduced the risk of a prespecified composite kidney outcome by 22% relative to placebo (HR 0.78; 95% CI 0.67–0.92), mainly driven by reduced incidence of new-onset persistent macroalbuminuria, with more limited effects on advanced kidney events [15]. Similarly, in SUSTAIN-6, semaglutide reduced the incidence of new or worsening nephropathy mainly through effects on macroalbuminuria [16]. These findings were the first consistent evidence that activating the GLP-1R might modify the natural history of diabetic kidney disease, though cardiovascular outcomes trials were not designed to evaluate kidney failure as a primary endpoint. Subsequent studies strengthened the evidence while introducing important distinctions between albuminuria-based and kidney function-based outcomes. In RE-WIND, dulaglutide reduced a composite kidney outcome comprising new macroalbuminuria, sustained eGFR decline, or kidney replacement therapy (HR 0.85; 95% CI 0.77–0.93), with the largest contribution arising from reduced macroalbuminuria [17]. Additional analyses suggested a favorable effect on more restrictive kidney function outcomes, although these analyses were not equivalent to a dedicated kidney outcome trial. The AWARD-7 study used an active comparator in patients with medium-to-advanced CKD and showed greater eGFR retention with dulaglutide than with insulin glargine at similar glycemic targets [18]. While this study design supports the idea that divergent kidney function was not solely due to achieving equivalent glycated hemoglobin levels, it does not rule out the role of other metabolic, hemodynamic, or therapy-related influences. In AMPLITUDE-O, efpeglenatide reduced a broad composite kidney outcome by approximately 32%, extending the evidence to an exendin-based GLP-1RA [19].

The FLOW trial marked a decisive transition from secondary kidney outcomes to a dedicated evaluation of clinically meaningful kidney protection. This double-blind, placebo-controlled trial enrolled 3533 patients with type 2 diabetes and albuminuric CKD, who were randomized to once-weekly semaglutide 1.0 mg or placebo beyond standard care [4]. The primary composite endpoint, sustained decline in kidney function, terminal kidney failure, or death due to kidney or cardiovascular causes, was decreased by semaglutide versus placebo by 24% (HR 0.76; 95% CI 0.66–0.88) with follow-up over a median of 3.4 years, translating to an absolute risk difference of 4.5%. The average kidney eGFR slope was 1.16 mL/min/1.73 m^2^ more adverse in the placebo arm versus the semaglutide arm. These findings provided convincing evidence that semaglutide can attenuate clinically relevant kidney disease progression in patients with established diabetic CKD, rather than merely reducing albuminuria. Several FLOW characteristics are particularly relevant to interpreting its mechanism. First, the trial enrolled patients with substantial baseline kidney risk, thereby increasing the likelihood of observing clinically meaningful changes in kidney function over the follow-up period. Second, the benefit was observed despite established protection from RAS inhibition in most participants. Thirdly, benefits were observed across several clinically relevant outcomes, increasing confidence that semaglutide alters disease trajectory. The primary endpoint was a composite that included a hard endpoint, cardiovascular death, which is less relevant to kidney disease but fails to disentangle the relative effects of cardiorenal protection per se and the pleiotropic effects of weight loss, modulation of endothelial function and systemic inflammation, and blood pressure lowering. Semaglutide has now been shown to provide clinical renoprotection, but the pathways driving this effect are not established.

Evidence from individuals without diabetes has expanded the conceptual framework of GLP-1RA-associated kidney protection. In SELECT, which enrolled 17,604 participants with overweight or obesity and established cardiovascular disease but without diabetes, semaglutide 2.4 mg reduced a prespecified composite kidney end-point (HR 0.78; 95% CI 0.63–0.96) [3]. This composite included kidney-related death, kidney replacement therapy, sustained severe kidney function impairment, a persistent reduction in eGFR of at least 50%, or incident persistent macroalbuminuria. Although the absolute event rate was relatively low, the findings suggested that semaglutide’s potential kidney benefits extend beyond populations with hyperglycemia. However, incident macroalbuminuria accounted for a large proportion of the composite endpoint, so the findings should not be interpreted as definitive evidence of a reduction in kidney failure risk among people without diabetes. The lack of diabetes does not exclude potential metabolic pathways, given that adjustments for adiposity, blood pressure, insulin resistance, and systemic inflammation may still have mediated treatment effects.

More direct evidence in non-diabetic CKD was provided by a randomized, double-blind trial involving 101 patients with overweight or obesity and albuminuric CKD without diabetes [5]. After 24 weeks, semaglutide 2.4 mg reduced UACR by 52.1% vs. placebo (95% CI 33.4–65.5%). This anti-proteinuric effect is impressive, given that the study included patients with several causes of kidney disease, such as chronic glomerulonephritis and hypertensive CKD. However, because the trial lasted only 24 weeks, it was underpowered to assess long-term effects on kidney function, and because albuminuria was the primary endpoint, no assessment could be made of kidney failure risk. These encouraging data justify testing GLP-1RAs in non-diabetic CKD. Efficacy by cause of kidney disease has not yet been demonstrated.

The SOUL trial provided additional evidence concerning the relationship between GLP-1RA treatment and kidney function trajectories. Among 9650 participants with type 2 diabetes and established atherosclerotic cardiovascular disease and/or CKD, oral semaglutide reduced major adverse cardiovascular events, while prespecified analyses assessed kidney outcomes [20]. The five-component composite kidney endpoint occurred in 8.4% of those treated with semaglutide and 9.0% of those receiving placebo (HR 0.91; 95% CI 0.80–1.05), which was not statistically significant. The kidney-specific composite excluding cardiovascular death was not much reduced (HR 0.86; 95% CI 0.66–1.10). The annual decline in eGFR slowed by about 0.40 mL/min/1.73 m^2^ compared with placebo. These differences highlight the influence of baseline kidney risk and the composition of event outcomes. Notably, participants in SOUL had better baseline kidney function than those in FLOW, and cardiovascular death accounted for a larger share of the broader composite outcome. The lack of a statistically significant reduction in kidney events should not be interpreted as evidence of no biological effect. However, the smaller difference in eGFR slope also suggests caution in assuming all groups will experience the same kidney benefits.

A prespecified pooled trial population-level analysis of SELECT, FLOW, and SOUL in 2026 further confirmed semaglutide’s beneficial effects on kidney outcomes within the cardiovascular kidney metabolic continuum [13]. In 30,787 participants, semaglutide lowered the risk of a broad composite of sustained major eGFR decline, kidney failure, kidney-related death, or cardiovascular death (HR 0.84, 95% CI 0.77–0.91). This composite was driven by the narrower composite excluding cardiovascular deaths, which semaglutide also favorably affected (HR 0.80, 95% CI 0.69–0.92). This matters because the latter composite reduces potential confounding by the overall effect observed in reductions in cardiovascular mortality. However, this analysis pooled populations with different risks of progressive kidney disease, different semaglutide doses, routes of administration, and background therapies. Also, combining several trials evaluating the same drug does not necessarily imply that equivalent effects are expected for all members of the GLP-1RA class. The results provide strong evidence for semaglutide’s kidney-protective potential across selected cardiometabolic populations, while leaving important questions about biological heterogeneity and treatment-response mechanisms unresolved.

Beyond average treatment effects, recent observational evidence suggests considerable heterogeneity in GLP-1RA-associated kidney protection. Xu et al. studied 28,547 patients with type 2 diabetes who initiated either GLP-1RAs or dipeptidyl peptidase-4 inhibitors. Initiation of GLP-1RAs was associated with a lower risk of composite kidney outcomes (HR 0.84; 95% CI 0.72–0.97). However, the estimated five-year absolute risk difference was −0.72 percentage points (95% CI −1.53 to 0.13), indicating a modest absolute benefit with uncertainty around the estimate. Importantly, patients in the highest quartile of kidney failure risk, assessed using the Kidney Failure Risk Equation, experienced a substantially greater absolute risk reduction (2.76%; 95% CI 0.08–5.60%) than those at lower baseline risk. Lower baseline eGFR, faster prior decline in kidney function, higher albuminuria, and higher glycated hemoglobin concentrations were associated with larger estimated treatment benefits. These signals indicate that established clinical indicators could be used to select patients at increased likelihood of experiencing significant absolute kidney benefits from GLP-1RA use. However, the inability to establish causality is limited by the observational nature, residual confounding, and external validation of the models used to estimate treatment effect. Also, a greater absolute benefit in high-risk patients does not necessarily mean biologically distinct patient groups differ in their responsiveness to treatment [21].

Albuminuria marks kidney damage and is an important prognostic marker, but a treatment-induced reduction in urinary albumin excretion does not necessarily reflect structural restoration of the glomerular filtration barrier. It may also result from modifications in intraglomerular pressure, endothelial permeability, podocyte integrity, tubular albumin reuptake, or some combination of these factors. Similarly, the eGFR slope offers critical insight into how renal function evolves but does not necessarily distinguish functional hemodynamic adaptations from structural retention of renal tissue. Changes in body composition can alter creatinine generation, which may obscure interpretation of creatinine-based eGFR during dramatic weight loss. For this reason, cystatin C-based measures or measured GFR may be particularly valuable tools for research on weight-loss therapies [22,23,24,25].

This difference between clinical benefit and biological causality is most relevant for whether the kidney benefit tied to GLP-1RA medication was independent of other systemic mechanisms of benefit. While randomization was sufficient to document the total causal effect of randomized treatment allocation on renal and cardiovascular clinical endpoints, it does not determine the nature or number of other systemic pathways that mediated the treatment benefit. For instance, changes in body weight, systemic hyperglycemia, blood pressure, inflammatory activity, and kidney function may not be independent, and adjusting for some, or even all, post-randomization variables does not necessarily establish a causal effect. Definitive characterization of direct intrarenal effects therefore requires additional evidence from human physiology, tissue-specific molecular analyses, receptor localization studies, and carefully designed mechanistic interventions [5,7,26,27].

## 4. Renal GLP-1 Receptor Biology and Hemodynamic Regulation

### 4.1. Renal GLP-1 Receptor Localization and Cellular Specificity

GLP-1R localization at the cellular level affects the biological plausibility of a direct kidney-protective effect. The receptor is part of the G protein-coupled receptor (GPCR) class B family of receptors, which generally couple to Gs-promoted stimulation of adenylyl cyclase, with resultant increases in the intracellular levels of cyclic adenosine monophosphate (cAMP) and subsequent downstream effectors like protein kinase A (PKA) and the exchange protein directly activated by cAMP (EPAC). The accepted signaling pathway does not necessarily imply physiologic downstream actions in a particular cell type. To show that receptor activation mediates cellular action, receptor expression, ligand-dependent signaling, and, ideally, the absence of the biological effect with targeted receptor blockade or genetic deletion are required. This is most relevant to the kidney, where systemic and local tissue responses vary across cell populations [7,28,29].

The precise anatomical location of GLP-1R expression in the human kidney has been highly controversial. Early experiments used immunohistochemistry and showed GLP-1R expression in proximal tubular epithelial cells, glomerular structures, and glomerular and extraglomerular vasculature. Follow-up work to identify methodological issues revealed cross-reactivity in several commercial GLP-1R antibodies and, as a result, raised questions about the previously observed tissue staining patterns. Pyke et al. then mapped GLP-1R localization in human and nonhuman primate tissues using an extensively validated monoclonal antibody [7]. Their work showed that the receptor localizes to smooth muscle cells of the renal artery and arteriole within the preglomerular vasculature, not throughout the renal tubular epithelium or the glomerular filtration barrier. Subsequent experiments using validated antibodies and receptor knockdown models confirmed the renal vascular compartment as a reproducible site of GLP-1R expression [30,31]. The data do not support the often-reported but not consistently proven direct, receptor-dependent effects of GLP-1RAs on all kidney cell types. Many in vitro studies show beneficial effects of GLP-1RAs on podocytes, mesangial cells, and proximal tubular epithelial cells, including reductions in oxidative stress, inflammation, apoptosis, and profibrotic phenotypic responses. These studies also do not provide conclusive evidence of GLP-1R expression and effect in vivo. Cell culture conditions may alter cell phenotype and receptor expression and may produce biological effects at drug concentrations much higher than the clinical dose. In addition, the lack of proper receptor-negative controls or receptor-specific antagonists, as well as receptor knockout/knockdown experiments, makes causality in current reports difficult to interpret. Accordingly, experimental evidence of improved podocyte or tubular cell function should be distinguished from definitive evidence of direct receptor activation within these compartments [7,28,29].

Recent advances in single-cell and single-nucleus transcriptomics offer opportunities to refine the cellular localization of renal GLP-1R. However, these technologies introduce new interpretative challenges. Low-abundance GLP-1R transcripts can make detection challenging. Although a single missed detection does not imply receptor absence, technical dropout, sequencing depth, and preferential cell recovery can affect whether detection is observed. Minimal receptor transcripts do not necessarily indicate sufficient receptor protein to support physiologically relevant signaling. Spatial transcriptomics provides anatomical context but lacks the resolution or sensitivity to distinguish deeply embedded vascular, endothelial, and juxtaglomerular cell populations. Consequently, a convincing characterization of human renal GLP-1R distribution will require orthogonal approaches integrating validated receptor-binding assays, spatially resolved transcript detection, protein localization, and functional signaling measurements [7,32,33,34].

Evidence for GLP-1R expression varies markedly across renal cell types, experimental species, and detection methods. There is relatively uniform evidence of GLP-1R expression in renal vascular smooth muscle cells from completed validated receptor-localization studies, but evidence for glomerular endothelial cells, podocytes, tubular epithelial cells, mesangial cells, and renal immune cells is less clear. Treatment-associated cellular responses are not a requirement of receptor expression per se, nor do they necessarily denote receptor-dependent signaling. Table 1 summarizes the available human and animal evidence, with receptor localization-based results in one column and functional data in another, and highlights the main areas of uncertainty.

The selective enrichment of GLP-1R in renal vascular smooth muscle cells is key to understanding the molecular signatures observed with treatment. The renal arterioles control the distribution of blood flow, glomerular capillary pressure, and the autoregulatory responses of the kidneys to systemic perfusion pressure. Experimental stimulation of vascular GLP-1R alters afferent arteriolar responsiveness and renal perfusion [30]. These studies suggest that the renal microcirculation may be a key anatomical site mediating the effects of GLP-1RAs on renal physiology. It should also be emphasized that vascular smooth muscle cells may influence the activity of the endothelial cells through changes in local perfusion, paracrine factors, the morphology of the adjacent ECM, and mechanical stimuli. This distinction matters when interpreting emerging human molecular evidence [9]. These findings align with an endothelial remodeling model of kidney protection, but do not prove that endothelial cells host the semaglutide receptor. Another possibility is that activating a semaglutide receptor in renal vascular smooth muscle cells, other extrarenal tissues, or another regulatory compartment induces secondary phenotypic changes in endothelial cells and immune cell recruitment. All of these pathways are hypothetically possible and not mutually exclusive. However, their relative contributions cannot be determined from transcriptomic associations alone.

Another complicating factor is species-specific differences in kidney and receptor distribution. While receptor knockout studies and renal autoregulation studies have helped elucidate GLP-1R mechanisms in rodents, differences in nephron number and structure, metabolic and vascular responsiveness, and renal injury disease models inherently limit translation to humans. GLP-1R expression and activity may also change in response to CKD-related conditions such as diabetes, obesity, inflammation, and renal injury. Such changes may impact receptor expression and responsiveness in humans, but this is not yet clearly established [7,37,38].

### 4.2. Tubuloglomerular Interactions and Renal Hemodynamics

Renal hemodynamic regulation is a biologically plausible but not fully elucidated mechanism by which GLP-1RAs may affect kidney disease progression. Glomerular filtration depends on the balance of renal plasma flow, glomerular capillary hydraulic pressure, oncotic forces, and the ultrafiltration coefficient, and autoregulatory mechanisms like the myogenic response and tubuloglomerular feedback co-regulate it, tending to preserve filtration pressure and rate despite changes in perfusion pressure. In diabetic kidney disease, altered proximal tubular sodium reabsorption, sodium chloride delivery to the distal tubule, and afferent arteriolar tone may influence the development of glomerular hyperfiltration and the mechanical stress burden on the filtration barrier. These factors are well established as nephropathy-related maladaptations in kidney injury pathophysiology, providing a meaningful physiological context for interpreting the renal effects of GLP-1RAs [37,39,40].

One of the most reproducible acute physiological responses to GLP-1 and GLP-1RAs is an increase in urinary sodium excretion. Experimental evidence suggests that alterations in the proximal tubule sodium-hydrogen exchanger isoform 3 (NHE3), a key player in proximal sodium-bicarbonate reabsorption, are involved. GLP-1 administration has been associated with reduced proximal tubule sodium reabsorption, along with changes in proximal tubule sodium handling, urinary pH, and fractional sodium excretion. However, the precise signaling pathway responsible for these effects remains uncertain, particularly given the limited evidence for functional GLP-1R expression in human proximal tubular epithelial cells. Proposed mechanisms include indirect regulation through renal vascular signaling, neurohumoral pathways, changes in renal perfusion, and interactions with the RAS. Therefore, the physiological demonstration of natriuresis should not be interpreted as definitive evidence of direct GLP-1R-dependent inhibition of tubular NHE3 [40,41,42].

Human mechanistic studies have provided particularly informative, although sometimes apparently conflicting, results. In a randomized, double-blind, placebo-controlled study of exenatide in patients with type 2 diabetes without renal impairment, Tonneijck et al. studied effects on urine Na^+^ and urine pH as indicators of proximal tubular sodium reabsorption, using inulin and para-aminohippurate clearance to quantify GFR and effective renal plasma flow, respectively [40]. Exenatide increased fractional excretion of urine Na^+^ and urine pH, consistent with a decreased ability of the proximal tubule to reabsorb Na^+^. This occurred without significant changes in measured GFR, effective renal plasma flow, filtration fraction, or estimated glomerular capillary hydrostatic pressure, showing that natriuresis with GLP-1RA can occur without measurable changes in global renal hemodynamics. Importantly, the absence of GFR reduction does not support the inference that GLP-1RAs replicate the innate hemodynamic response of SGLT2 inhibition. SGLT2 inhibitors inhibit proximal sodium-glucose co-transport, increase distal sodium chloride delivery, and can “reset” tubuloglomerular feedback, leading to an early GFR decline. GLP-1RAs could all affect proximal sodium transport. However, downstream effects on renal vascular resistance, neurohormones, and distal sodium delivery may differ. The coupling of macula densa sodium delivery with afferent arteriolar constriction is modulated by the baseline renal function, proximal tubule reabsorptive function, extracellular volume, and renal autoregulatory precision, so that natriuresis alone does not explain a similar pattern of intraglomerular pressure reduction [37,40,43,44].

The complexity of GLP-1RA-associated renal hemodynamic effects is further illustrated by investigations in individuals without diabetes and in a small physiological study involving healthy overweight men. Exenatide administration increased measured GFR and effective renal plasma flow, accompanied by reduced estimated afferent arteriolar resistance and increased estimated glomerular hydraulic pressure. Pharmacological inhibition of nitric oxide synthase attenuated several of these responses, suggesting that nitric oxide-dependent vascular mechanisms contributed to the observed hemodynamic effects. However, unlike the lack of significant changes in GFR and renal plasma flow in patients with type 2 diabetes [42], the results differ. Potential explanations for this discrepancy may include differences in renal physiology, metabolic status, vascular responses to adrenergic stimulation, and experimental procedures. As with clearance-based estimates, glomerular capillary pressure calculations rely on physiological assumptions and do not directly measure intraglomerular pressure.

Experimental investigations have also implicated GLP-1R signaling in renal vascular autoregulation. Jensen et al. showed that activating GLP-1R in renal vascular smooth muscle cells altered afferent arteriolar autoregulatory responses and increased renal blood flow in experimental preparations [30]. These observations link receptor localization to functional vascular responses. However, the consequences of altered autoregulation for long-term kidney protection remain uncertain. Increased renal perfusion may improve microvascular oxygen delivery under some conditions, but in others, attenuation of afferent arteriolar autoregulatory constriction could theoretically increase glomerular capillary pressure. The direction and clinical significance of hemodynamic responses cannot be inferred from receptor activation alone.

The renin–angiotensin–aldosterone system (RAAS) represents another potential mediator of GLP-1-associated renal responses. Human physiological studies have reported suppression of circulating angiotensin II during GLP-1 administration under certain experimental conditions, whereas other studies have found limited or inconsistent effects on systemic renin and aldosterone concentrations [38]. GLP-1RA administration can induce *renin* gene expression in renal vascular smooth muscle cells in mice [45]. Although these observations suggest interactions between GLP-1R signaling and intrarenal renin regulation, their physiological significance remains uncertain. In particular, changes in *renin* gene expression within vascular smooth muscle cells should not automatically be equated with increased systemic renin activity or activation of the circulating RAS. Instead, these effects may reflect local vascular adaptation or altered cellular differentiation within the renal arterial tree.

An important unresolved question is whether acute changes in sodium transport and renal perfusion meaningfully contribute to the long-term preservation of kidney function observed in clinical outcome trials. Acute clearance studies typically involve small populations, short exposure periods, and participants with relatively preserved kidney function, whereas the FLOW trial demonstrated clinically meaningful benefits over several years in patients with established albuminuric CKD [4]. So, the physiological effects shown with exenatide administration should not necessarily be assumed to mediate the long-term kidney benefits of semaglutide.

The cross-talk between SGLT2 inhibitors and GLP-1RA can affect proximal tubule sodium handling, but they seem to have different acute renal hemodynamic profiles. Their combined effects might operate through partially complementary mechanisms rather than through a shared additive effect. SGLT2 inhibition is well characterized in its relationship with tubular sodium-glucose transport and tubuloglomerular feedback, whereas GLP-1RA-associated effects may involve a broader range of vascular, neurohumoral, metabolic, and inflammatory responses. These conclusions require adequately powered randomized comparisons and mechanistic studies that specifically evaluate combination therapy [13,46,47].

Beyond increasing glomerular filtration, changes in regional perfusion distribution may also affect tissue oxygenation and injury. The kidney is physiologically predisposed to be sensitive to alterations in local oxygen balance because tubular sodium transport is an oxygen-consuming process that occurs at a high demand, and oxygenation is limited by its unique local vascular architecture. Because of these vulnerabilities, human studies have described GLP-1-dependent regulation of renal cortex and medullary perfusion [38], but this does not necessarily imply improved tissue oxygenation, because oxygen supply and demand can be altered simultaneously. Attenuation of renal hypoxia should be independently confirmed by direct measures of renal oxygenation, tubular workload, and tissue microcirculation.

## 5. Molecular Mechanisms of Kidney Protection: From Endothelial Dysfunction to Fibrosis

### 5.1. Glomerular Endothelial Remodeling and Microvascular Protection

The glomerular microvasculature is proposed to mediate the kidney-protective effects of GLP-1RAs. Traditionally, diabetic kidney disease has been considered a disease of podocyte injury, mesangial expansion, and glomerular basement membrane thickening. However, mounting evidence supports the concept that endothelial dysfunction is an early, potentially amplifying, component of progressive glomerular injury. Glomerular endothelial cells provide a specialized fenestrated vascular interface and regulate the filtration barrier via their glycocalyx, local inflammatory responses, and cell–cell cross-communication with glomerular podocytes, mesangial cells, and circulating immune cells. Loss of function in these cell types may increase albumin permeability, attract leukocytes, promote endothelial-to-mesenchymal signaling, and drive progressive glomerulosclerosis. This suggests that therapeutic approaches that restore endothelial homeostasis may be renoprotective, regardless of whether they normalize tubular or podocyte function [48,49,50]. The endothelial glycocalyx may be a more relevant interface of metabolic stress with glomerular hyperpermeability. The glycocalyx is a negatively charged, carbohydrate-rich layer lining the endothelium that extends the molecular barrier to dynamic alterations in vascular permeability, mechanotransduction, and helps preserve an anti-inflammatory state of the endothelium. Hyperglycemia, reactive oxygen species (ROS), altered shear stress, and inflammatory mediators can all contribute to glycocalyx degradation, facilitating albumin translocation and leukocyte adhesion. However, direct improvement of the glomerular endothelial glycocalyx has not been demonstrated as a mechanism underlying the renal effects of GLP-1RAs, even though all endothelial dysfunction determinants have improved [48,50,51].

The recently published REMODEL trial provides the most direct human tissue-based evidence supporting glomerular endothelial involvement in semaglutide-associated kidney protection [9]. In this trial, 106 individuals with type 2 diabetes and CKD were randomized 1:1 to semaglutide 1 mg or placebo once weekly for 52 weeks. They combined multiparametric renal MRI with kidney biopsy, single-nucleus RNA sequencing (snRNA-seq), and spatial transcriptomics. Semaglutide failed to meet the coprimary imaging endpoints of renal oxygenation, global perfusion, and tissue inflammation using MRI-derived metrics. This negative primary result must remain central to the study’s interpretation. However, secondary analyses showed a reduction in the renal artery resistive index and stabilization of the apparent diffusion coefficient, also indicating reduced alteration in fibrotic tissue. These image changes suggest potential benefits in renal vessel physiology and tissue remodeling, but do not specifically relate to glomerular capillary pressure or histological fibrosis. The molecular analyses revealed a particularly pronounced response in glomerular endothelial cell populations.

In the single-nucleus RNA sequencing analysis, only two glomerular endothelial clusters showed substantial transcriptional changes that may be linked to treatment. Genes differentially expressed in the treatment groups within the glomerular endothelial cell clusters were tied to metabolic stress, inflammatory signaling, vascular function, and ECM regulation, including genes related to transforming growth factor beta (TGF-β) signaling, NF-κB, phosphoinositide 3-kinase/Akt signaling, and cellular response to mechanical stress. Expression of genes tumor necrosis factor receptor superfamily member 14 *(TNFRSF14*), fms-related tyrosine kinase 4 (*FLT4*), and mitogen-activated protein kinase 8 interacting protein 3 (*MAPK8IP3*) was decreased with semaglutide treatment within the glomerular endothelial cell populations. These data are consistent with blunted endothelial stress-related transcriptional pathways but do not directly indicate down-regulation of those pathways at the protein level, nor do they demonstrate the functional relevance of specific gene expression changes. Another strength of REMODEL was its integration of spatial transcriptomic information. Spatial analyses suggested fewer immune cells near glomerular endothelial populations after semaglutide treatment, while computational ligand-receptor analyses identified changes in predicted endothelial–immune communication networks. Signaling interactions between platelet and endothelial cell adhesion molecule 1 (PECAM1) and vascular endothelial cadherin, as well as between integrins and ECM ligands, linked these responses. These data support the hypothesis that semaglutide alters the glomerular microenvironment to promote endothelial junctional integrity and reduce inflammatory interactions. However, computationally inferred ligand–receptor communication is a prediction based on gene expression, not direct experimental evidence of functional intercellular signaling [9,52].

Well-characterized receptor-localization studies show GLP-1R expression predominantly in renal vascular smooth muscle cells, with scant evidence of extensive functional receptor expression in glomerular endothelial cells. Thus, the endothelial transcriptional profiles in REMODEL might reflect indirect effects of altered vascular resistance, mechanical forces, systemic metabolic abnormalities, or paracrine signals from receptor-expressing cells. This interpretation offers a biologically coherent explanation for how a relatively restricted distribution of renal GLP-1R could generate broader molecular responses across the glomerular microvascular compartment [7,9,30].

**Figure 1:**
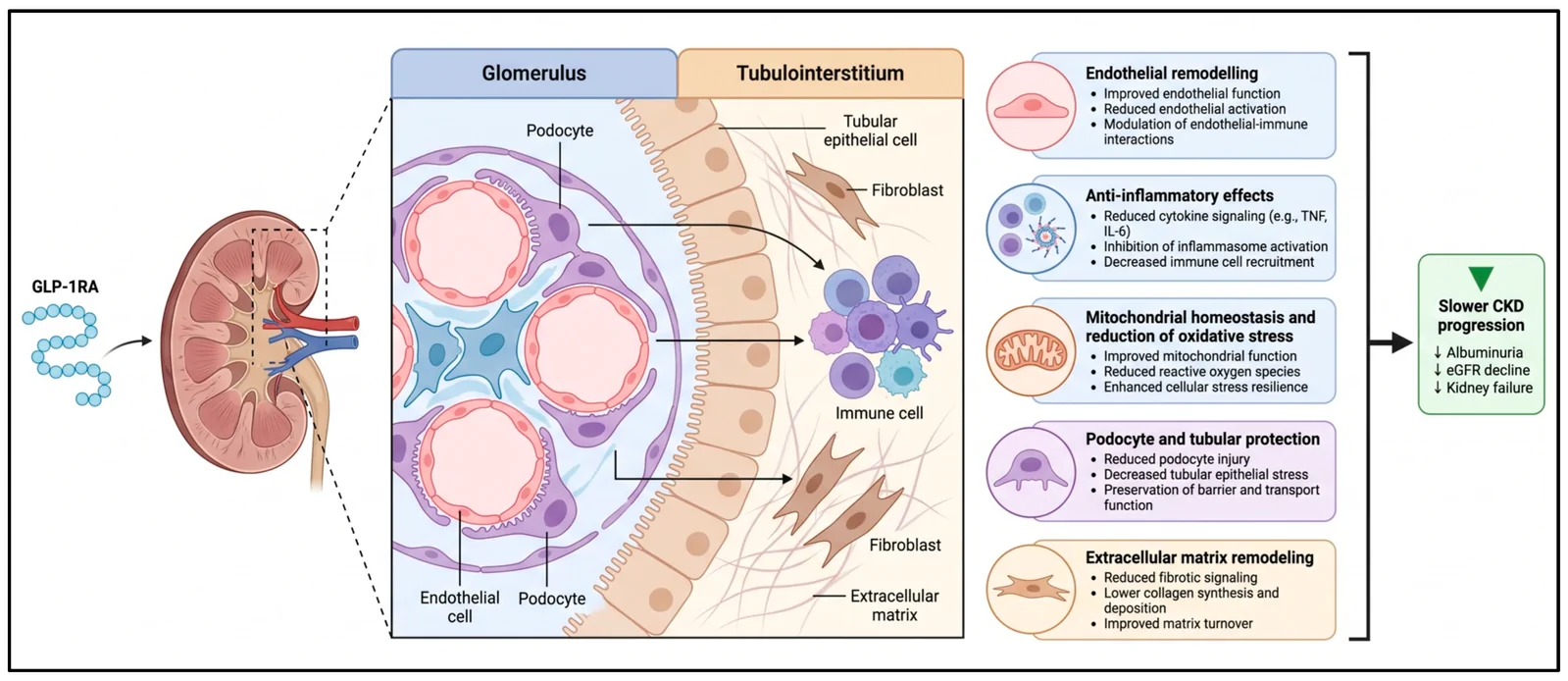
Proposed molecular mechanisms of glucagon-like peptide-1 receptor agonist (GLP-1RA)-related kidney protection. Illustration of pathways that may be interconnected in a complex network, including improvements in glomerular endothelial cells, inflammation, and mitochondrial function; podocyte and tubular cell protection; and extracellular matrix remodeling. These processes are thought to suppress albuminuria and slow CKD progression. These pathways are based on a mixture of experimental data, preliminary human molecular analysis, and mechanistic interpretation. Uncertainty remains as to whether glomerular endothelial cells, podocytes, and tubular epithelial cells all express GLP-1R, and it remains to be determined how the individual pathways influence kidney protection.

The communication channel between glomerular endothelial cells and podocytes through soluble factors, ECM interaction, and mechanotransduction is well known. Higher endothelial barrier function may affect podocyte stress and glomerular permeability, even without direct GLP-1R activation on podocytes. The time course of endothelial transcriptome changes related to albuminuria reduction and renoprotection is poorly understood, and causality remains to be elucidated. We will likely need non-invasive assessments of endothelial function over time, spatial molecular proteomics, and well-characterized biomarkers of glomerular injury to distinguish whether endothelial remodeling mediates renoprotection or merely reflects a healthier kidney, as in reductions in renal inflammation [7,9]. The potential links between GFR decline, albuminuria, GLP-1RA-induced glomerular endothelial remodeling, inflammation, mitochondrial function, podocyte and tubular protection, and ECM remodeling are depicted in Figure 1.

### 5.2. Inflammation, Oxidative Stress, and Mitochondrial Dysfunction

An imbalance in the generation and detoxification of ROS describes a chronic low-grade inflammation and oxidative stress imbalance that leads to further deterioration of the diabetic kidney, obesity, and CKD. Increased metabolic substrate generation, altered mitochondrial electron transport, increased advanced glycation end products (AGEs), engagement of inflammatory receptors, and reduced antioxidant capacity impair cell function. This contributes to glomerular barrier and tubulointerstitial injury affecting the endothelium, podocytes, tubular epithelium, resident macrophages, and interstitial fibroblasts. The potential anti-inflammatory effects of GLP-1RAs are of significant scientific interest, especially because reductions in albuminuria and maintenance of kidney function may involve biological pathways beyond changes in glomerular hemodynamics [53,54].

NF-κB is a key transcriptional mediator that couples metabolic stress to inflammatory gene expression. NF-κB activation may elicit a range of changes, including increased cytokine and chemokine production, increased adhesion molecule expression, and the recruitment of inflammatory cells. In experimental models of diabetic kidney disease, GLP-1RA treatment has often been linked to dampened NF-κB-associated inflammatory signals. This dampening may be secondary to improved glucose regulation and/or insulin resistance, body fat distribution, or oxidative stress. In addition, suppressing NF-κB target gene expression does not necessarily mean directly suppressing NF-κB transcriptional activity. The REMODEL study provides important evidence from human tissue supporting changes in inflammatory pathways, mainly in glomerular endothelial and immune cell populations, but it does not confirm a receptor-dependent molecular pathway linking semaglutide exposure to NF-κB inhibition [9].

The NLRP3 inflammasome adds another potential rigid link between metabolic derangement and renal inflammation. Inflammasome activation favors caspase-1-dependent cleavage of interleukin (IL)-1 and IL-18 and may contribute to a vicious circle of inflammatory perpetuation and pyroptotic cell death. Preclinical research indicates that GLP-1RAs may modulate inflammasome-related signaling within metabolically deranged tissues. However, it remains to be established whether inflammasome inhibition explains an improvement in human CKD, with *NLRP3* gene induction, inflammasome formation, caspase-1 cleavage, and mature cytokine production representing discontinuous effects that cannot be substituted for each other. As a result, inflammasome blocking must be demonstrated through readouts of individual pathways, coupled with receptor-dependent techniques [55,56,57].

Oxidative stress commonly drives inflammation, mitochondrial damage, and renal dysfunction. Oxidative stress links inflammation, mitochondrial damage, and decreased renal function. Excess ROS production is connected to lipid peroxidation, protein oxidation, DNA damage, endothelial dysfunction, and stimulation of profibrotic signals. Nicotinamide adenine dinucleotide phosphate (NADPH) oxidases and the mitochondrial electron transport system are major candidate sources of ROS in metabolically impaired renal tissue. GLP-1RA-associated improvements in oxidative stress markers have been reported in several experimental models, but the interpretation of these findings is complicated by the close relationship between oxidative injury and systemic metabolic control. In addition, commonly used measures of oxidative stress, including malondialdehyde concentrations and total antioxidant activity, lack the cellular and pathway specificity needed to define a mechanism of kidney protection [54,58,59,60].

Recent studies have identified ferroptosis as a potentially relevant mechanism linking altered lipid metabolism, iron handling, and oxidative tubular injury. Ferroptosis is a regulated form of cell death characterized by iron-dependent accumulation of phospholipid hydroperoxides and failure of cellular antioxidant defense systems. In experimental models, semaglutide was associated with changes in β-Klotho expression, iron homeostasis, lipid peroxidation, and antioxidant capacity, along with attenuated inflammatory and fibrotic responses [61]. These observations suggest that metabolic regulation of ferroptotic susceptibility may contribute to tissue protection. However, several limitations constrain the interpretation of this mechanism. First, β-Klotho does not directly activate the GLP-1R in tubular epithelial cells. Second, while decreases in lipid peroxidation and cellular iron content are consistent with ferroptosis inhibition, they should be confirmed by functional rescue experiments and pathway-specific interventions. Third, the relative contribution of ferroptosis to kidney disease progression in humans receiving GLP-1RAs has not been determined. Accordingly, ferroptosis should currently be considered an emerging experimental hypothesis rather than an established mechanism of clinical renoprotection [61,62,63,64].

An additional potential target is mitochondrial dysfunction. Renal tubule epithelial cells, mostly proximal tubule cells, largely depend on mitochondrial oxidative metabolism to generate adenosine triphosphate (ATP) necessary for transport function. Changes in mitochondrial substrate utilization, respiratory chain activity, redox state, and organelle homeostasis might contribute to tubule damage and incomplete recovery after an insult. Preclinical data show that GLP-1RA treatment favorably modulates mitochondrial bioenergetics and apoptosis signaling under metabolic stress. For example, exenatide and exendin-4 have been associated with improved mitochondrial function and altered energy metabolism in experimental diabetic kidney injury models [65]. These findings support a potential relationship between GLP-1-associated metabolic adaptation and cellular resilience. Improved mitochondrial function cannot necessarily be interpreted as mitochondrial-directed effects. GLP-1RAs can, directly or indirectly, affect substrate supply, systemic insulin sensitivity, lipid metabolism, and cellular energy status. Mitochondrial membrane potential, ATP content, oxidative stress, and respiratory chain capacity reflect different aspects of mitochondrial function, and mechanisms should separate mitochondrial function from mitochondrial amount, substrate consumption, and cellular workload [28,54,66,67]. The interaction between mitochondrial function and quality-control mechanisms must also be considered. Although autophagy and mitophagy degrade proteins and organelles, abnormal autophagy often increases ROS and cytokine generation. AMP-activated protein kinase, mammalian target of rapamycin, and other downstream effectors in cell-protective pathways are involved in GLP-1RA. Upregulation of autophagy-effector proteins does not imply increased autophagic flux. Definitive mechanistic interpretation requires dynamic assessment of autophagosome formation and degradation, preferably combined with pathway-specific perturbation [68,69,70,71].

Another important consideration is emerging evidence that dual incretin receptor agonists may act through multiple pathways. In studies of mice treated with tirzepatide, diabetic kidney damage was also less severe, and oxidative stress and PI3K/Akt signaling were affected [72]. Because tirzepatide dual-activates the glucose-dependent insulinotropic polypeptide and GLP-1Rs, these studies do not provide evidence of GLP-1R signaling effects. Future head-to-head comparisons between selective GLP-1RAs and dual agonists may clarify receptor-specific mechanisms but must match pharmacodynamics and pharmacokinetics.

### 5.3. Podocyte Injury, Cellular Senescence, and Fibrotic Remodeling

Podocyte injury and fibrotic remodeling are important determinants of irreversible nephron impairment. Podocytes preserve the structural and functional integrity of the glomerular filtration barrier through their specialized actin cytoskeleton, slit diaphragm proteins, and interactions with the glomerular basement membrane. Because post-mitotic podocytes have limited replacement capacity in adults, sustained cellular stress leads to foot process effacement, detachment, reduced podocyte numbers, and secondary glomerular sclerosis. Metabolic problems, mechanical strain, mitochondrial dysfunction, inflammatory mediators, and altered endothelial–podocyte communication may all contribute to these processes [49,73,74,75].

Experimental studies have reported that GLP-1RAs attenuate several features of podocyte injury, including apoptosis, oxidative stress, cytoskeletal abnormalities, and changes in slit diaphragm-associated proteins. However, interpretation of these findings is complicated by uncertainty regarding functional GLP-1R expression in podocytes. Consequently, improved podocyte survival following GLP-1RA treatment should not automatically be interpreted as a direct receptor-mediated effect. Indirect mechanisms, such as reduced glomerular pressure, improved endothelial function, altered circulating inflammatory mediators, or improved systemic metabolic conditions, may offer alternative explanations [7,35,36].

By changing the ECM composition, paracrine signaling, local oxygen delivery, and inflammatory cell recruitment, endothelial damage can affect podocyte activity. Conversely, podocyte-generated mediators support glomerular capillary integrity and endothelial differentiation. Loss of this crosstalk may critically aggravate filtration barrier failure. The endothelial transcriptional changes identified in REMODEL suggest that semaglutide-associated podocyte protection may arise partly through restoring glomerular microvascular homeostasis rather than direct podocyte receptor activation [9]. This hypothesis is mechanistically attractive but requires experimental validation using multicellular glomerular systems and cell-specific receptor perturbation.

Cell senescence may also contribute to renal failure. Senescent cells exhibit persistent cell-cycle arrest and metabolic abnormalities, including mitochondrial dysfunction and inflammatory signaling. The senescence-associated secretory phenotype can drive local tissue inflammation, matrix protein accumulation, and cellular dysfunction. Chronic metabolic and oxidative stress can drive podocytes and tubular epithelial cells into senescence. GLP-1RA-induced improvements in mitochondrial function and inflammatory signaling could attenuate cell senescence or its development. However, evidence that GLP-1RAs directly reverse established senescence in human kidney tissue remains limited [76,77,78]. This distinction matters because decreases in individual senescence markers do not demonstrate reversal of the senescent phenotype. Markers like p16, p21, senescence-associated β-galactosidase, and inflammatory cytokines are not individually specific. Even changes in cellular stress responses may make a cell appear senescent without persisting cell-cycle arrest. A future approach might involve assessing a panel of markers together with functional assessment of the senescence-associated phenotype and cell-specific molecular phenotyping [79,80].

Fibrotic remodeling represents the end-stage in many progressive renal diseases. It is induced by sustained injury leading to activation of different fibrotic cell types (including interstitial fibroblasts) and induction of matrix-producing cell populations. As a consequence, ECM production increases, the organ’s architecture breaks down, and renal function is lost. Several pathways control fibrogenesis, with TGF-β/SMAD and mediators of inflammation, mechanical force, stretch, cell metabolism, and cell-ECM interaction being particularly relevant. Most experimental data link GLP-1RAs to downregulation of TGF-β, collagen, and fibronectin expression, as well as fibroblast activation markers. However, changes in these markers do not necessarily establish reduced net matrix accumulation or reversal of established fibrosis [81,82,83]. The REMODEL trial offers preliminary human evidence relevant to this question. Semaglutide treatment was associated with stabilization of the renal apparent diffusion coefficient and changes in transcriptional programs related to fibrotic signaling [9]. These data are consistent with reduced tissue remodeling, but diffusion-weighted MRI is sensitive to biological and technical factors, including cellularity, perfusion, and water mobility. The apparent diffusion coefficient cannot be used as a fibrosis-specific metric. Similarly, variation in TGF-β-associated transcripts does not specifically indicate collagen production and deposition, matrix breakdown, or fibroblast activity.

Another challenge is distinguishing inhibition of new ECM synthesis from enhanced degradation of the existing matrix. These processes may have different biological consequences and may not be adequately captured by conventional kidney function measurements. Circulating and urinary collagen fragments, including markers of type III collagen turnover, may provide complementary information about ECM dynamics. However, interpreting them requires considering systemic collagen metabolism, kidney clearance, and the relative contributions of the glomerular and tubulointerstitial compartments [84,85,86].

The association of glomerular injury and tubulointerstitial fibrosis should not be overlooked. Persistent albuminuria may trigger proximal tubular cellular stress and associated inflammatory signaling that can spill over, causing tubulointerstitial injury. Interventions that reduce albumin filtration by strengthening the glomerular filter could indirectly exert anti-inflammatory and antifibrotic effects on tubulointerstitial tissue. Conversely, systemic metabolic improvements may independently influence tubular substrate utilization and inflammatory activity. These possibilities suggest that apparently distinct glomerular and tubulointerstitial responses may be causally connected rather than representing separate pharmacological effects [87,88,89].

These responses still need not necessarily be initiated by de novo receptor activation on all cell types involved. Instead, relatively restricted receptor expression may initiate vascular or systemic adaptations that subsequently influence broader intrarenal signaling networks [7,9]. A critical next step will be to establish the timing and causal impact of these responses. Human longitudinal studies, renal imaging, tissue transcriptomics and spatial proteomics, and novel biomarkers of ECM turnover can investigate whether these receptor-dependent changes in endothelial cell phenotype are accompanied by podocyte stabilization and decreased progression of fibrotic scar formation. Experimental approaches that include cell-specific GLP-1R knockout, receptor-selective pharmacology, and multicellular kidney tissue models are needed to distinguish primary GLP-1R-dependent effects from secondary tissue adaptations [9,30,52,90].

The central question is no longer whether semaglutide can reduce kidney disease progression in selected patients, but which biological processes mediate this protection, whether these processes differ across CKD phenotypes, and whether their activity can be quantified using molecular biomarkers. Answering these questions will be essential to translating emerging mechanistic evidence into individualized, kidney-protective treatment strategies [4,9]. The proposed individual molecular mechanisms behind GLP-1RA kidney protection as discussed here have varying levels of evidence. While clinical studies have established kidney-protective effects, the specific roles of each molecular pathway remain under investigation. Evidence from in vivo animal and in vitro studies helps elucidate these mechanisms, but further research is needed to define receptor-mediated effects and their applicability to humans. Table 2 summarizes the evidence available from human (clinical), animal, and in vitro studies for the mechanisms summarized in this review.

## 6. Translating Molecular Mechanisms into Biomarkers

### 6.1. Biomarkers of Glomerular, Tubular, and Fibrotic Responses

The current markers, UACR and eGFR, will likely remain the fundamental features for predicting clinical benefit and CKD progression. Nevertheless, both will not exactly meet the energetic demand to have enough biological information on which cellular pathways have been directly shifted by the intervention. This is relevant for the renoprotective effects of GLP-1RAs, which could orchestrate systemic vascular inflammatory and fibrotic mechanisms. In this way, a translational combination of universal clinical response parameters and system-based molecular biomarkers might define the biological impact of GLP-1R activation on the kidney [1,4,9]. Candidate biomarkers for assessments of GLP-1RA-related kidney benefit and their clinical validation status are summarized in Table 3. While albuminuria is an existing clinical biomarker and circulating TNFR1/2 show prognostic relevance to CKD progression, both lack evidence as predictive biomarkers of GLP-1RA renal benefit. Many other molecular markers, including endothelial markers, collagen turnover markers, and kidney tissue transcriptional signatures, remain exploratory and lack independent validation.

Prognostic, predictive, and pharmacodynamic biomarkers differ in important ways: prognostic biomarkers indicate the risk of disease progression regardless of treatment. Predictive biomarkers indicate the relative benefit of treatment, which varies by the treatment administered. Pharmacodynamic biomarkers change with treatment exposure and may not be causally related to outcomes, provide proof of mechanism, or offer unambiguous evidence of treatment benefit. These concepts are not synonymous or interchangeable. In particular, a biomarker that changes substantially in a patient receiving GLP-1RA treatment cannot be assumed to mediate kidney benefit or predict treatment response. Establishing these relationships requires appropriate longitudinal sampling, randomized treatment comparisons, formal interaction analyses, and ideally independent validation [98].

Albuminuria represents the most extensively investigated kidney biomarker in GLP-1RA trials. Several agents, including liraglutide, dulaglutide, and semaglutide, have consistently reduced UACR, and this reduction is an important component of the kidney benefits reported in cardiovascular outcome studies. More recently, the FLOW trial demonstrated that semaglutide also reduces clinically meaningful kidney events and attenuates eGFR decline in patients with type 2 diabetes and established CKD [4]. However, the mechanisms underlying reduced albuminuria are biologically diverse. Albumin excretion rates in urine could be caused by changes in intraglomerular pressures, endothelial cell barrier integrity, podocyte viability, and proximal tubule albumin processing. That means UACR is an established marker of kidney injury and therapy response but does not determine the mechanism of GLP-1RA-associated renoprotection. This limitation is particularly relevant to the emerging hypothesis that glomerular endothelial remodeling contributes to semaglutide-associated kidney protection. The REMODEL trial demonstrated treatment-associated changes in glomerular endothelial transcriptional programs and spatial relationships between endothelial and immune cell populations [9]. In particular, these results suggest that biomarkers of endothelial activation, vascular inflammation, and ECM interactions could offer insight beyond albuminuria. Candidate markers include angiopoietin-2, vascular adhesion molecules, and markers of endothelial glycocalyx degradation. However, these are not kidney-specific, and plasma levels may reflect overall vascular disease, inflammation, adiposity, and heart disease. Their use should be assessed in kidney-specific molecular signatures rather than taken as a direct marker of glomerular endothelial damage.

Tubular injury biomarkers may provide complementary information because glomerular and tubulointerstitial responses are closely interconnected. A key candidate is kidney injury molecule-1 (KIM-1), a transmembrane protein highly upregulated in injured proximal tubular epithelial cells. KIM-1 concentrations in the circulation and urine have been tied to kidney injury and adverse kidney outcomes in various clinical situations. Other candidate markers include neutrophil gelatinase-associated lipocalin (NGAL), liver-type fatty acid-binding protein (LFABP), and certain inflammatory mediators. Interpret them with caution, as some markers are strongly affected by acute tubular injury, systemic inflammation, or kidney clearance, and their value for monitoring chronic GLP-1RA treatment effects remains poorly defined [94,95,99,100].

Recent proteomic studies have further clarified cellular responses to GLP-1RA use. In a post hoc analysis of the AWARD-7 trial, McFarlin et al. studied the change at six months of 21 circulating proteins (measured as part of the Joslin Kidney Panel) in 249 patients with type 2 diabetes and moderate-to-severe CKD treated with dulaglutide (*n* = 124) or insulin glargine (*n* = 125) [93]. They identified 14 proteins that differed significantly between treatment arms, with concentrations increasing with insulin glargine and decreasing with dulaglutide. The most prominent differences were observed among eight members of the tumor necrosis factor receptor (TNFR) superfamily, TNFR1 and 2, known mediators of inflammatory and apoptotic signals. Additional treatment-associated differences were observed for cluster of differentiation (CD)160, WAP four-disulfide core domain 2 (WFDC2), Delta-like canonical Notch ligand 1 (DLL1), layilin (LAYN), syndecan-1 (SYND1), and EPH receptor A2 (EPHA2). Conversely, KIM-1 decreased in both arms with no between-group difference. Proteomic changes were especially significant in those with lower baseline eGFR or higher baseline albuminuria, hemoglobin A1c (HbA1c), or body mass index (BMI). These data suggest that GLP-1RA treatment may modulate circulating inflammatory and fibrotic protein signatures beyond kidney-specific biomarkers. Given the exploratory nature of this analysis, the small study population, and lack of tissue validation, these data cannot prove a causal effect on pathway blockade or elucidate how these pathways mediate long-term kidney benefits.

TNFRs are particularly interesting because circulating levels have been consistently associated with progression of diabetic kidney disease. TNFR1 and TNFR2 likely reflect inflammatory activity, injury, and disrupted receptor shedding, and they are also influenced by kidney function and general health. Variation in circulating TNFR levels associated with preserved kidney function could reflect changes in inflammatory activity, protein production or breakdown, or both. Showing that changes in circulating levels explain preserved kidney function would require time-dependent analyses that link biomarker changes to subsequent kidney outcomes [26,92,101,102].

Biomarkers of turnover are particularly attractive because they link molecular mechanisms to the structural progression of kidney disease. Renal fibrosis is characterized by a dynamic imbalance in ECM synthesis, degradation, and remodeling, not just collagen deposition. Biomarkers for different components of collagen metabolism should complement one another. Researchers have investigated serum PRO-C6, a marker associated with type VI collagen formation, and urinary C3M, a fragment generated during type III collagen degradation, in patients with diabetic CKD receiving dulaglutide [97]. In an exploratory analysis of AWARD-7 involving 330 participants, dulaglutide was associated with lower serum PRO-C6 concentrations and higher urinary C3M concentrations compared with insulin glargine. These changes were interpreted as potentially reflecting reduced collagen formation and altered extracellular matrix degradation, respectively.

Serum PRO-C6 measures collagen turnover outside the kidney, and urinary C3M may reflect multiple processes, including local matrix degradation, tubular handling, and urinary biomarker excretion. Increased collagen degradation fragments do not necessarily indicate a reduction in total fibrotic burden. The balance between matrix synthesis and degradation, together with the spatial distribution of remodeling, determines the structural consequences of these processes [84,85,103].

A more recent DC-Ren study further supports the potential pharmacodynamic relevance of urinary C3M [104]. Among 229 participants with type 2 diabetes, combined RAS inhibition and GLP-1RA treatment was associated with a more favorable longitudinal urinary C3M trajectory than RAS inhibition alone. Lower baseline urinary C3M levels were also linked to a higher risk of disease progression. The data imply that products of ECM breakdown may reveal information about tissue remodeling caused by treatment, which is not detected by albuminuria or eGFR. Although the treatment comparison is observational and the sample size is modest, the study provides no direct histological confirmation of antifibrotic effects.

### 6.2. Multi-Omics and Molecular Phenotyping

With improved access to high-dimensional molecular technologies, we can move beyond biomarker research to more globally characterize GLP-1RA kidney responses. Proteomic, metabolomic, transcriptomic, and spatially resolved tissue-based molecular profiling can capture multiple dimensions of tissue biology and reveal coordinated alterations in renal endothelial function, immune activation, tubular metabolism, and ECM remodeling. The relevance of these approaches hinges not only on measuring large numbers of molecular features, but on their ability to form robust, biologically interpretable, and clinically meaningful signatures [9,12,105,106].

Rather than examining only circulating markers, the research also assessed treatment response in kidney tissue. This resolution to single cells is mostly relevant to describing tissue anatomy, as alterations in small cell populations can be missed in bulk tissue measurements.

Single-nucleus RNA sequencing measures transcript abundance rather than protein expression or pathway activity, and differences in cell composition may influence apparent treatment-associated changes. Spatial transcriptomic approaches provide anatomical information but may have limited sensitivity for low-abundance transcripts and cannot independently establish functional cell-to-cell communication. Furthermore, REMODEL’s molecular analyses were based on relatively small subsets of participants with paired biopsy samples, increasing the potential influence of sampling variability and limiting statistical power [9].

The differential regulation of 14 Joslin Kidney Panel proteins following dulaglutide treatment suggests that coordinated changes in circulating inflammatory and fibrosis-associated proteins may provide pharmacodynamic information beyond conventional clinical endpoints [93]. However, it remains unclear whether these signatures predict subsequent kidney outcomes or identify patients with greater relative treatment benefit. Their clinical application will require longitudinal validation, integration with established kidney risk markers, and demonstration of incremental predictive value.

Proteomics profiling offers an alternative approach that could be more easily scaled to large clinical trials. Circulating proteins reflect multiple processes, including inflammation, vascular injury, ECM remodeling, and metabolic regulation. An exploratory proteomic analysis described in 2026 used SELECT samples and assessed more than 6300 proteins in roughly 11,000 patients taking semaglutide or placebo [96]. Over 350 proteins were linked to kidney-related outcomes, including KIM-1, TNFR1, collagen XVIII alpha-1 chain, matrix metalloproteinase-7 (MMP-7), and natriuretic peptide B. Approximately 1400 proteins exhibited treatment-related alterations, with a smaller percentage coinciding with recognized potential kidney biomarkers. Among these, angiopoietin-2, natriuretic peptide B, and collagen XVIII alpha-1 chain were both prognostic and therapy-responsive. This may be of interest because it suggests that semaglutide’s molecular responses map to networks involved in vascular dysfunction, ECM biology, and cardiovascular-renal crosstalk. However, the results are currently available only as a conference abstract and must be corroborated by a full peer-reviewed article. Also, treatment response and prognostic association do not imply predictive capability. A biomarker may change with semaglutide treatment and independently predict CKD progression without identifying patients who derive greater relative treatment benefit. Therefore, formal treatment-by-biomarker interaction analyses, external replication, and assessment of incremental predictive performance are necessary before such signatures can be considered clinically actionable [12,107,108].

Metabolomics may further clarify how systemic metabolic adaptation affects kidney protection. GLP-1RAs, by increasing satiety and reducing caloric intake, body weight, and adiposity, and by improving glucose homeostasis and possibly altering substrate utilization, may change the metabolite profile in blood and urine. Putative mechanisms may include lipid oxidation, amino acid pathways, tricarboxylic acid cycle metabolites, acylcarnitines, and oxidative stress-related molecules, but these are most likely markers of dietary intake and metabolism outside the kidney. During large weight loss, correcting nutritional intake and muscle mass, and the resulting substrate availability to body tissues, may also affect circulating metabolite levels and kidney function [109,110].

Urinary extracellular vesicles provide an alternative, but technically challenging, source of material. Vesicles shuttle proteins, lipids, and nucleic acids from nephron and urothelium cells and may therefore reflect kidney-related molecular processes without repeated tissue biopsies. Analyzing vesicles shed into the urine could then enable assessment of membrane transporter pathways, cellular stress responses, and cell-to-cell signaling pathways influenced by GLP-1RAs. However, vesicle purification techniques, normalization methods, non-vesicular contamination, and questions about cellular origin all impede reproducibility. For this reason, urinary extracellular vesicles should be viewed as an exploratory application rather than a validated technique for monitoring renal protection with GLP-1RAs [111,112,113].

Circulating biomarkers provide a system-wide view. Urinary biomarkers may reflect kidney injury and/or excretion, and kidney tissue cells and structure serve as the final reference. Their combined analysis could help distinguish systemic treatment effects from intrarenal molecular responses. For example, concordant changes in circulating inflammatory proteins, glomerular endothelial transcriptional programs, and urinary markers of ECM turnover would strengthen the biological plausibility of coordinated anti-inflammatory and antifibrotic effects. However, concordance alone would not establish causal mediation, particularly when several biomarkers are influenced by kidney clearance or common upstream metabolic changes [9,26,114].

A translational approach to biomarker-guided GLP-1RA therapy would therefore include a series of synergistic steps. First, discovery will require testing reproducible molecular signals across multiple randomized treatment pairs and, ultimately, validating them in independent studies using a shared sampling and measurement approach. Candidate biomarkers must show a relationship with the most relevant kidney outcomes, such as durable loss of eGFR and kidney failure, not changes in albuminuria alone. Finally, benefit prediction will rely on a baseline profile’s ability to modify the magnitude of treatment benefit, while pharmacodynamics will require that therapy-induced changes reliably indicate a well-defined biological process. When mechanistic mediation is proposed, longitudinal measurements and appropriate causal analytical frameworks are needed to distinguish mediators from treatment-related consequences [12,26,91,107].

Patients with comparable eGFR and albuminuria levels may vary widely in endothelial activation, inflammatory load, tubular stress, or extracellular matrix remodeling. It is not yet clear whether these molecular variations affect the magnitude of benefit from GLP-1RAs. Recent tissue-based evidence from REMODEL, exploratory proteomic findings from SELECT, and pharmacodynamic collagen biomarker studies provide a rationale for investigating this possibility [9,96,114].

**Figure 2:**
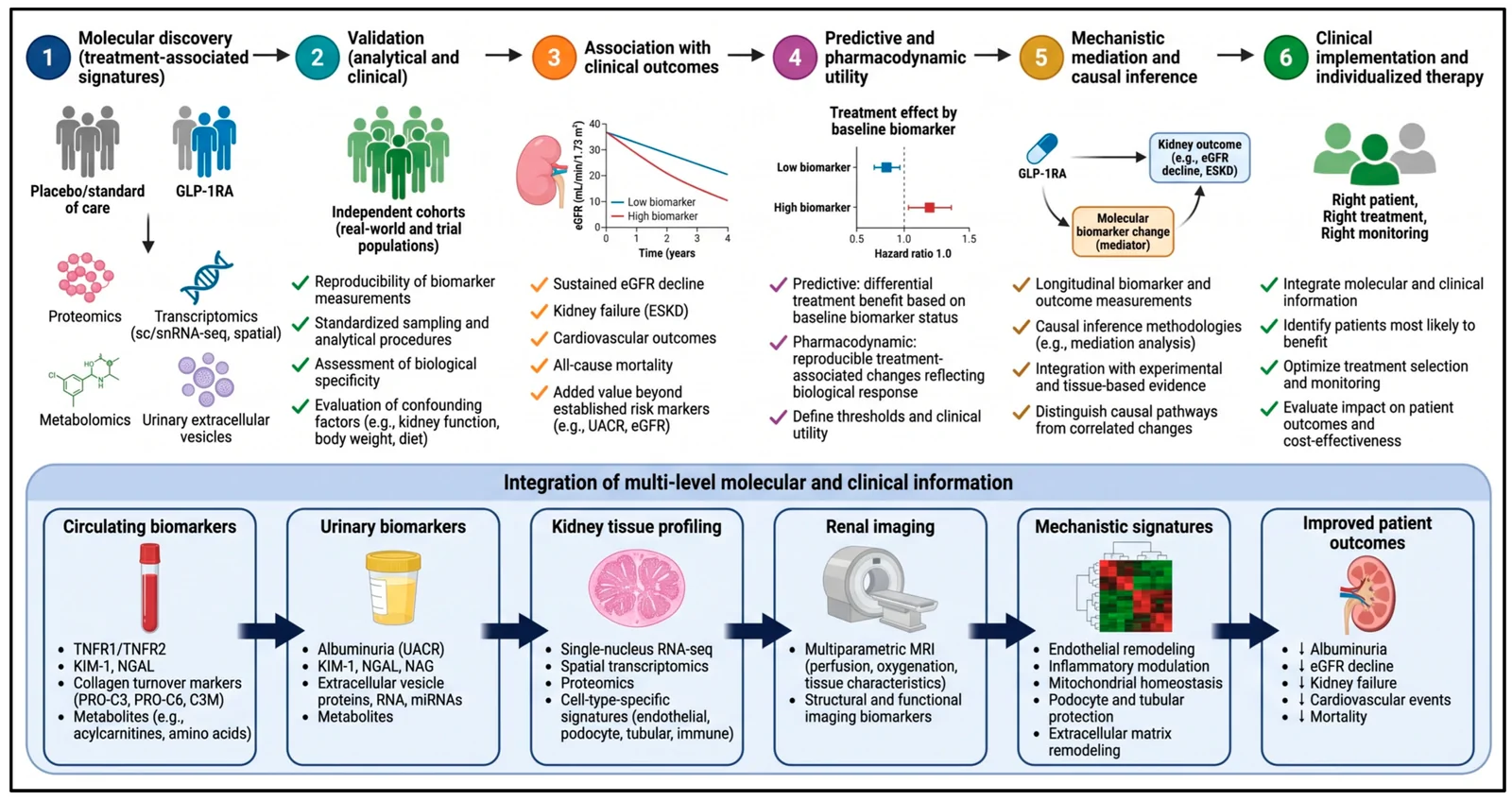
Integrated translational approach to molecular phenotyping and biomarker-guided glucagon-like peptide-1 receptor agonist (GLP-1RA) therapy in chronic kidney disease. The scheme shows how circulating and urinary biomarker signatures, kidney tissue transcriptomics and proteomics, metabolomics, and imaging can be integrated into a molecular phenotyping setup to define treatment-associated molecular signatures. Candidate molecular signatures must be analytically validated and independently replicated. Derive and validate biomarkers that predict clinically meaningful kidney outcomes in predictive models, then use them to quantify the incremental prognostic value of predictive markers relative to traditional clinical risk markers. Causal effects along the treatment pathway must be validated using longitudinal data and experimental models. Ultimately, molecularly guided treatment selection should show incremental clinical utility over traditional clinical risk markers. This is a conceptual schematic; no biomarker-guided GLP-1RA treatment approach has been clinically validated.

Molecular phenotyping may help elucidate the biological processes underlying changes in conventional kidney biomarkers, such as albuminuria and eGFR. However, applying novel molecular biomarkers clinically requires demonstrating analytical validity, biological specificity, prognostic or predictive value, and incremental utility beyond established kidney risk markers. By associating molecular responses with randomized treatment effects and long-term kidney outcomes, upcoming investigations will reveal whether GLP-1RA-related renal protection can be concisely summarized by reproducible and specific molecular signatures and can be translated into personalized treatment plans [91,98,107,114]. An integrated translational framework linking molecular discovery, cross-compartment biomarker profiling, analytical and clinical validation, and mechanistic investigation to the potential development of individualized GLP-1RA treatment strategies is presented in Figure 2.

## 7. Conclusions

The FLOW trial established that semaglutide reduces clinically meaningful kidney outcomes, while complementary evidence from SELECT, SOUL, and subsequent pooled analyses has expanded our understanding of its potential benefits across different cardiometabolic populations [3,4,13,20]. Still, we remain largely uncertain how well we can target these mechanisms. Our greater challenge now is to move from understanding that we can deliver clinical benefit to understanding the cellular mechanisms underlying kidney protection and to quantify, track, and intentionally target them.

A crucial difference to consider when making clinical inferences from these results is whether the effects observed are specific to semaglutide or can be generalized to the GLP-1RA class more broadly. While several GLP-1RAs have produced positive cardiovascular and renal outcomes, differences in molecular structure, pharmacological properties, meaning of outcomes, populations studied, and clinical trials may mean that we should not necessarily apply outcomes from these agents to all other GLP-1RAs. In particular, the renal outcomes benefit from FLOW and the exploratory molecular effects shown in REMODEL (affecting endothelial inflammation, mitochondria, and fibrosis) should be specific effects of semaglutide alone, rather than representing class effects that we can assume to be true of all GLP-1RAs.

At a more detailed level, an important question that remains to be resolved is the anatomical localization and functional significance of renal GLP-1R expression. Despite support from these receptor-localization studies, definitive evidence of broad receptor expression in podocytes, tubular epithelial cells, and glomerular endothelial cells remains limited [7,30,31]. In particular, changes in inflammatory signaling, mitochondrial biology, or ECM metabolism after a GLP-1RA do not necessarily imply direct action of a circulating or locally expressed receptor in the affected cells. Future studies will need to develop valid methods to localize receptor expression, including cell-specific receptor genetic perturbations, receptor-targeted drugs, and measurements of cell-specific GLP-1R downstream signaling, possibly using human kidney organoid and multicellular glomerular models. However, the limits of these models should be acknowledged.

A second challenge is establishing the causal relationship between renal vascular responses and structural tissue protection. Acute physiological studies have demonstrated GLP-1RA-associated natriuresis, but effects on glomerular filtration rate, renal plasma flow, and estimated intraglomerular pressure vary across populations and experimental conditions [38,40,45]. These observations indicate that GLP-1RAs’ long-term benefits cannot currently be explained by a uniform hemodynamic response analogous to that observed with SGLT2 inhibitors. Longitudinal studies that integrate measured kidney function, renal perfusion imaging, tubular transport assessments, and molecular biomarkers could clarify whether early physiological changes predict subsequent structural preservation.

Its findings of treatment-related changes in glomerular endothelial transcriptional programs and endothelial–immune cell interactions also substantiate the potential importance of microvascular remodeling. More generally, the absence of benefit on the coprimary imaging endpoints, combined with the exploratory nature of tissue-based analyses and the small cohort size, precludes firm mechanistic interpretation. Follow-up investigations should focus on replicating such tissue findings in an independent population with particular attention to the need for longitudinal tissue profiling and for experimental confirmation of potential candidate pathways within the endothelium, especially to distinguish GLP-1R-dependent effects on endothelial signaling from secondary endothelial changes induced by alterations in systemic metabolism, renal blood flow, or inflammation.

Another question remains: the causality of mechanisms such as inflammation, oxidative stress, cell senescence, fibrotic remodeling, and mitochondrial dysfunction. Preclinical studies suggest that NF-κB signaling, inflammasome activation, ferroptosis, mitochondrial housekeeping, and ECM regulation may be mechanisms by which GLP1 RA exerts benefit [8,53,61,65,71,82]. However, most of these have been evaluated only in preclinical models and may not be causally related to CKD in humans. In addition, increases in individual molecular markers may reflect reduced cellular stress rather than inhibition of a specific pathway. That means rigorous demonstrations should include pathway perturbation, rescue of organ function by pathway activation, and time-resolved molecular measures rather than relying on correlations between gene and protein expression.

Developing clinically informative biomarkers is a fourth major priority. Conventional measures such as the UACR and eGFR remain essential for evaluating kidney outcomes but provide limited insight into the biological mechanisms underlying treatment responses. Recent proteomic and extracellular matrix biomarker studies have identified potentially relevant changes in tumor necrosis factor receptors, inflammatory proteins, and collagen turnover markers during GLP-1RA treatment [93,97,104]. Nevertheless, no molecular biomarker has yet been validated to identify patients who derive greater relative kidney benefit from these therapies. Future studies must distinguish prognostic biomarkers from pharmacodynamic indicators and genuine predictors of treatment response, using randomized treatment comparisons, prespecified interaction analyses, and independent validation [98].

An additional priority is to determine whether molecular responses differ across CKD phenotypes. Most available robust evidence of hard clinical outcomes has been obtained in people with type 2 diabetes and albuminuric CKD. At the same time, the biological effects of GLP-1RAs in those with non-diabetic CKD, non-albuminuric disease, and more advanced levels of kidney impairment remain incompletely defined [3,4,5]. The degree to which kidney protection depends on baseline metabolic abnormality, obesity, endothelial activation, inflammation, or fibrosis remains unknown, as does the potential complementary benefit of GLP-1RAs with SGLT2 inhibitors and nonsteroidal mineralocorticoid receptor antagonists. Although these interventions affect at least partly distinct pathways, biological complementation does not necessarily translate into additive effects on the risk of kidney failure or other outcomes.

This uncertainty warrants a research system of RCTs with human kidney tissue, molecular phenotyping, and experimentally validated causal models. In particular, we suggest that long-term RCTs could clarify whether an initiating endothelial and inflammatory phenotype can be distinguished from subsequent insults such as albuminuria, tubular injury, ECM turnover, and further deterioration of kidney function. Once long-term RCTs are in place, this temporal information could differentiate initiating from secondary phenotypes. Spatial transcriptomics, proteomics, metabolomics, and noninvasive imaging, combined with clinical validators, could provide a more detailed understanding of the biological mix that affects treatment responses. Ultimately, the clinical usefulness of these strategies will hinge on their analytical reproducibility, biological specificity, additional predictive value, and cost-effectiveness in standard clinical settings [9,26,114].

The therapeutic use of GLP-1RAs has expanded well beyond glucose control to a novel and increasingly important role as a kidney-protective therapy in selected CKD populations. Newer evidence points towards the possibility that these benefits may be imparted through a coordinated shift in renal vascular regulation, glomerular endothelial homeostasis, inflammatory signaling, cellular metabolism, and the remodeling of the ECM. In this context of co-regulated molecular and cellular responses, recent studies in human tissue have drawn particular attention to glomerular endothelial remodeling, though its causal role in kidney protection remains to be elucidated.

## Figures and Tables

**Table 1:** Evidence for cell-specific GLP-1 receptor expression in human and experimental kidneys.

Renal Cell Type	Human mRNA Evidence	Human Protein Evidence	Animal mRNA Evidence	Animal Protein Evidence	Overall Interpretation	References
Renal vascular smooth muscle cells	Not independently established by the cited studies	GLP-1R localized to renal arterial and arteriolar smooth muscle using a validated monoclonal antibody	Renal vascular receptor expression supported experimentally; cell-specific transcript evidence requires further confirmation	Receptor localization and functional vascular responses demonstrated in experimental studies	Relatively strong evidence for renal vascular GLP-1R expression and function	[7,30,31]
Glomerular endothelial cells	No convincing cell-specific GLP1R transcript evidence established in the cited studies	No consistent receptor localization using validated antibodies	Not established	Not established	Direct endothelial GLP-1R expression remains uncertain; semaglutide-associated transcriptional changes do not demonstrate receptor expression	[7,9,30]
Podocytes	Not established	No convincing widespread receptor localization with validated antibodies	Not established by the cited experimental studies	Not conclusively established	Liraglutide-associated podocyte protection has been reported, but direct receptor-mediated signaling remains uncertain	[7,35,36]
Proximal tubular epithelial cells	No consistent cell-specific evidence established	Earlier immunohistochemical reports suggested expression, but antibody specificity and reproducibility remain concerns	Not conclusively established	Reported in experimental literature, but the cited sources do not establish reproducible cell-specific localization	Direct tubular GLP-1R signaling remains controversial	[7,28,29]
Mesangial cells	Not established	No convincing validated localization	Not established	Not established	Reported functional responses do not independently establish direct receptor expression	[7,28,29]
Renal immune cells	Not established in kidney-resident immune cell populations	No definitive kidney-specific localization	Not established	Not established	Treatment-associated changes in immune-cell activity may be indirect; renal immune-cell GLP-1R expression requires investigation	[9,28]
Interstitial fibroblasts	Not established	No validated evidence of widespread receptor expression	Not established	Not established	Antifibrotic treatment effects do not establish fibroblast-specific receptor signaling	[7,28,29]

Evidence classifications reflect the studies cited in this review and should not be interpreted as a systematic exclusion of receptor expression. “Not established” indicates that convincing cell-specific evidence was not identified in the cited studies, rather than definitive absence of expression. Functional responses to GLP-1RAs are separate from receptor localization. Detecting transcripts does not necessarily mean the receptor protein is functional, and low transcript levels may be missed due to technical issues. To be confident of receptor localization, specific validated antibodies or binding approaches, proper negative controls, and ideally cell-specific functional validation are needed. Abbreviations: GLP-1R, glucagon-like peptide-1 receptor; GLP-1RA, glucagon-like peptide-1 receptor agonist; mRNA, messenger RNA.

**Table 2:** Summary of evidence supporting proposed molecular mechanisms of GLP-1RA-mediated kidney protection.

Proposed Mechanism	Human Clinical Studies	Animal Models	In Vitro Experiments	References
Renal hemodynamic regulation	Altered natriuresis and variable renal perfusion responses	GLP-1R-dependent vascular autoregulatory responses	Limited direct evidence	[30,40]
Glomerular endothelial remodeling	REMODEL: altered endothelial transcriptional signatures; negative coprimary imaging endpoints	Experimental evidence supports vascular involvement	Endothelial signaling responses reported	[9,30]
Anti-inflammatory signaling	Exploratory changes in inflammatory transcriptional programs	Reduced inflammatory signaling in experimental kidney injury models	Modulation of NF-κB and inflammasome-associated pathways	[9,61]
Oxidative stress and mitochondrial function	Limited direct mechanistic evidence	Improvements in oxidative stress and mitochondrial-associated pathways	Changes in mitochondrial function and autophagy-associated markers	[65]
Podocyte protection	No definitive demonstration of direct receptor-mediated protection	Reduced experimental podocyte injury	Reduced apoptosis and cytoskeletal injury	[35,36]
Cellular senescence and fibrosis	No definitive evidence of reversal in human kidney tissue	Experimental modulation of injury and fibrosis-related pathways	Changes in senescence and profibrotic markers	[61,65]

Abbreviations: GLP-1RA, glucagon-like peptide-1 receptor agonist; GLP-1R, glucagon-like peptide-1 receptor; NF-κB, nuclear factor kappa B. Evidence from experimental models does not necessarily establish direct receptor-mediated effects in human kidney tissue.

**Table 3:** Candidate biomarkers for evaluating GLP-1RA-associated kidney protection.

Domain/Biomarker	Potential Biological Information	Key Interpretive Limitations	Key Interpretive Limitations	References
Albuminuria (UACR)	Glomerular filtration barrier function and kidney risk	Established clinical biomarker: widely validated for CKD risk assessment and monitoring treatment response	Changes may reflect hemodynamic effects or tubular albumin handling rather than structural repair	[4,91]
Circulating TNFR1/TNFR2	Inflammation-associated risk of CKD progression	Prognostically validated; exploratory for GLP-1RA response: associated with kidney disease progression, but treatment-response utility remains unvalidated	Concentrations depend on production, receptor shedding, and renal handling; do not directly demonstrate TNF pathway activity	[92,93]
KIM-1, NGAL and other tubular injury markers	Tubular epithelial injury and cellular stress	Clinically investigated; exploratory for GLP-1RA response: associations with kidney injury and outcomes, but limited treatment-specific validation	Limited specificity for individual drug mechanisms; incremental prognostic value varies	[93,94,95]
Endothelial-associated proteins (e.g., ANGPT2)	Systemic vascular and endothelial-associated treatment responses	Exploratory: treatment-associated proteomic findings require independent confirmation	Circulating concentrations are not glomerulus-specific; SELECT proteomic findings remain exploratory	[96]
Collagen turnover markers (PRO-C3, PRO-C6, C3M)	Extracellular matrix formation and degradation processes	Exploratory: preliminary clinical treatment-response associations without established clinical utility	Changes in collagen fragments do not independently establish reduced tissue fibrosis or regression of established fibrosis	[84,85,97]
Kidney tissue endothelial transcripts	Cell-type-resolved transcriptional programs associated with treatment	Early exploratory: tissue-based molecular findings from REMODEL, without independent clinical validation	Small biopsy subsets; transcript abundance does not establish protein activity, functional pathway modulation, or causal mediation	[9]

Clinical validation status refers to the maturity of evidence for the indicated biomarker and its proposed clinical application. Established prognostic utility does not imply validation as a predictive biomarker of GLP-1RA treatment benefit. None of the exploratory molecular biomarkers listed has been established for routine selection or monitoring of GLP-1RA therapy. Abbreviations: ANGPT2, angiopoietin-2; CKD, chronic kidney disease; GLP-1RA, glucagon-like peptide-1 receptor agonist; KIM-1, kidney injury molecule-1; NGAL, neutrophil gelatinase-associated lipocalin; TNFR, tumor necrosis factor receptor; UACR, urinary albumin-to-creatinine ratio. PRO-C3 and PRO-C6 reflect collagen formation; C3M reflects type III collagen degradation.

## Data Availability

No new data were created or analyzed in this study. Data sharing is not applicable to this article.

## Abbreviations

The following abbreviations are used in this manuscript:

AMPK	Adenosine monophosphate-activated protein kinase
ANGPT2	Angiopoietin-2
ATP	Adenosine triphosphate
BMI	Body mass index
cAMP	Cyclic adenosine monophosphate
C3M	Matrix metalloproteinase-generated type III collagen degradation fragment
CD	Cluster of differentiation
CKD	Chronic kidney disease
CI	Confidence interval
DLL1	Delta-like canonical Notch ligand 1
DNA	Deoxyribonucleic acid
ECM	Extracellular matrix
eGFR	Estimated glomerular filtration rate
EPAC	Exchange protein directly activated by cAMP
EPHA2	EPH receptor A2
FLT4	Fms-related receptor tyrosine kinase 4
GPCR	G protein-coupled receptor
GFR	Glomerular filtration rate
GLP-1	Glucagon-like peptide-1
GLP-1R	Glucagon-like peptide-1 receptor
GLP-1RA	Glucagon-like peptide-1 receptor agonist
HbA1c	Hemoglobin A1c
HR	Hazard ratio
IL-18	Interleukin-18
KIM-1	Kidney injury molecule-1
LAYN	Layilin
LFABP	Liver-type fatty acid-binding protein
MAPK8IP3	Mitogen-activated protein kinase 8 interacting protein 3
MMP-7	Matrix metalloproteinase-7
MRI	Magnetic resonance imaging
NADPH	Nicotinamide adenine dinucleotide phosphate
NF-κB	Nuclear factor kappa B
NGAL	Neutrophil gelatinase-associated lipocalin
NHE3	Sodium–hydrogen exchanger isoform 3
NLRP3	NOD-like receptor family pyrin domain-containing 3
PECAM1	Platelet and endothelial cell adhesion molecule 1
PI3K	Phosphoinositide 3-kinase
PKA	Protein kinase A
PRO-C3	Type III collagen formation biomarker
PRO-C6	Type VI collagen formation biomarker
RAAS	Renin–angiotensin–aldosterone system
RAS	Renin–angiotensin system
RNA	Ribonucleic acid
ROS	Reactive oxygen species
SANRA	Scale for the Assessment of Narrative Review Articles
SGLT2	Sodium–glucose cotransporter-2
snRNA-seq	single-nucleus RNA sequencing
SYND1	Syndecan-1
TGF-β	Transforming growth factor beta
TNFR	Tumor necrosis factor receptor
TNFR1	Tumor necrosis factor receptor 1
TNFR2	Tumor necrosis factor receptor 2
TNFRSF14	Tumor necrosis factor receptor superfamily member 14
UACR	Urinary albumin-to-creatinine ratio
WFDC2	WAP four-disulfide core domain 2
